# Rumination and Emotional Profile in Children with Specific Learning Disorders and Their Parents

**DOI:** 10.3390/ijerph17020389

**Published:** 2020-01-07

**Authors:** Paola Bonifacci, Valentina Tobia, Vanessa Marra, Lorenzo Desideri, Roberto Baiocco, Cristina Ottaviani

**Affiliations:** 1 Department of Psychology, University of Bologna, Viale Berti Pichat, 5, 40127 Bologna, Italy; vanessa.marra@live.it (V.M.); lorenzo.desideri2@unibo.it (L.D.); 2 Faculty of Psychology, Vita-Salute San Raffaele University, via Olgettina, 58, 20132 Milano, Italy; tobia.valentina@hsr.it; 3 Department of Developmental and Social Psychology, Sapienza University of Rome, via dei Marsi, 78, 00185 Rome, Italy; Roberto.Baiocco@uniroma1.it; 4 Department of Psychology, Sapienza University of Rome, Sapienza University of Rome, via dei Marsi, 78, 00185 Rome, Italy; cristina.ottaviani@uniroma1.it; 5 Neuroimaging Laboratory, Santa Lucia Foundation, via Ardeatina 306, 00142 Rome, Italy

**Keywords:** rumination, specific learning disorders, emotional profile, family

## Abstract

Rumination, namely a cognitive process characterized by a repetitive thinking focused on negative feelings and thoughts, is a significant predictor for the onset of internalizing symptoms and has also been found to run in families. Rumination has never been studied in children with specific learning disorders (SLD), a population that, due to its condition, might encounter more difficulties in daily life and is at risk of increased psychological distress, compared to typically developing (TD) peers. The present study covers this gap by examining whether children with SLD, and their parents, tend to use rumination more than TD peers and their parents. The study also explores associations between rumination and both children’s and parents’ emotional profile. Results on 25 children with SLD and 25 TD peers and their parents (*n* = 150), showed higher levels of rumination in children with SLD when referring to a negative social situation, as well as higher levels of rumination in both mothers and fathers of children with SLD. Modest correlations between parents’ and children’s rumination traits were also found. This study offers evidence on rumination as a possible risk factor for children with SLD, particularly considering when they deal with social contexts.

## 1. Introduction

Specific learning disorders (SDL), i.e., neurodevelopmental disorders that impede learning or efficiently using reading, writing, or math skills [1] have an ascertained neurobiological etiology, nowadays best explained as deriving from multiple risk factors [2], that involve genetic, cognitive, and environmental influences [3]. Due to the difficulties that people encounter in their daily lives, particularly in the scholastic/academic settings, longitudinal evidence suggests that, as a consequence of dealing with their condition, students with SLD might develop increased psychological distress [4] compared to typically developing (TD) children. Notably, it has also been found that parents of children with SLD have increased parental stress compared to parents of TD children [5,6]. Recently, rumination, defined as a cognitive process characterized by an abstract, repetitive style of thinking focused on negative feelings and thoughts and their consequences, emerged to be a significant predictor for the onset of internalized disorders [7]. Children with SLD (and their families) might experience, more often than their TD peers, negative events in the school context (e.g., school failures, bad marks), in the family context (e.g., stressful child–parents interaction) or in the social context (e.g., difficulties with peers). To the best of our knowledge, however, no previous study assessed rumination in SLD populations to examine whether these children use rumination as a dysfunctional way to cope with such negative events. The first aim of this study was to cover this gap by examining whether children with SLD tend to use rumination more than TD controls. In light of recent studies pointing to an association between rumination in children and the dispositional tendency to use such coping strategy in their parents [8,9,10], we also aimed at exploring parent–child relationships in ruminative thinking and its emotional correlates.

In the following section, we briefly review significant literature on rumination and its emotional correlates in children with SLD and relationships between parents’ and children’s emotional profile. 

### 1.1. Rumination and Its Emotional Correlates in Children with SLD

A wide literature showed that students with SLD often exhibit internalizing symptoms, such as anxiety and depression [11,12] and found that these symptoms are increased by comorbid disorders [13]. Both in children and in adults, such internalizing symptoms are associated with rumination (see [14] for a recent review), which is particularly supposed to become a risk factor for the development of psychopathological symptoms during adolescence via poor self-regulation [15]. Indeed, Nolen-Hoeksema conceptualized rumination as a trans-diagnostic factor [16], which, although being mainly associated with depression [17,18], represents a risk factor for multiple forms of psychopathology including anxiety in adults and young people [19,20].

Importantly, the tendency to use rumination as a coping style has been found to increase with age, likely due to the amplified pressure that the school environment has on children as they grow [21,22]. For this reason, the early identification of ruminative processes in children is particularly important to prevent the development of a stable ruminative style in the later stages of development. Rumination in the developmental age has received little scientific attention, although it represents a particularly relevant variable for the prevention of discomfort and the promotion of well-being in children [23]. The perceived difficulties in managing potential threatening events, such as reading aloud may be for children with SLD, are a major source of anxiety [24,25]; consistently, higher mean scores on measures of emotional arousal and anxiety have been found [26]. For example, Tobia et al. [27] observed atypical skin conductance activation during a reading-aloud task that correlated with parents’ reports of emotional difficulties in dyslexic children, and the authors suggested that the same blunted autonomic response has been observed during worry (e.g., [28]). Rumination is difficult to inhibit and depletes the ability to exert attentional control (e.g., [29]), for this reason, students with SLD, who often have associated attentional symptoms (e.g., [30]), may be at higher risk for difficulties in inhibiting ruminative thinking. 

Children with SLD also show lower scholastic and interpersonal self-esteem [6,31], and low self-concepts of reading abilities could be associated with anxiety symptoms, emotional withdrawal, and passivity (refs. [32,33]; see also [34] for results on writing), as well as with difficulties in peer relationships [35]. However, supporting relationships and interactions in educational settings might represent key factors in students with SLD to shift from a prior negative self-perception to a current positive social identity [36]. Thus, rumination in children with SLD may occur not only with reference to negative events within the scholastic setting but also in relation to peer acceptance and family relationships. A particularly useful tool to investigate rumination in specific contexts, such as at school, with peers and with their family, is the Kid Rumination Interview (KRI; ref. [8]) which includes vignettes and cartoons for a children-friendly evaluation of this cognitive process in different prototypical situations that are well-suited for the aim of the present study. For this reason, the KRI has been used to assess rumination in children with SLD and controls.

### 1.2. Relationships between Parents’ and Children’s Emotional Profile

Studies on parents of children with SLD found higher worries about their children’s future [37], increased parental stress and different parenting styles (higher laxity and over-reactivity) compared to parents of children with typical development [5,6]. Thus, being a parent of a child with neurodevelopmental disorders might challenge the perception to fulfill these demands adequately and, therefore, might increase the psychological cost of parenthood. However, parents of children with SLD do not always show higher levels of depression, or negative functioning or other psychopathological indexes [6,38]. This suggests that having a child with SLD might have a significant impact on the parental role, but that parents of children with SLD are not per se characterized by emotional disturbances; individual parents’ psychological well-being is assumed to be a broader and multifactorial construct. On the other hand, based on the literature on family-risk studies [39], parents of children with SLD might themselves have weaknesses at the cognitive levels, often in the same areas of those manifested by their offspring, according to the broader phenotype constructs [40]. This, in turn, might amplify their perception of being ineffectual in their parental role, particularly if such parents did not receive appropriate intervention and support. 

Psychological profiles of parents have been found to have a reciprocal interaction with children’s psychological well-being. For example, it has been suggested that anxious and depressive symptoms are correlated with mistrust of parents [41], whereas feeling in tune with parents (the mother in particular) is considered a primary protective factor in children with SLD [42]. Furthermore, there are many evidences of intergeneration transmission of self-regulation between parents and children, in different developmental age, from infancy to school-age up to adolescence (for a review see [43]). 

Even more relevant to the aim of the present study, rumination has also been found to run in families. The Response Styles Theory [44], suggests that parents play an important role in the development of a ruminative style in children, highlighting that rumination is a stable and enduring cognitive style that is modeled during childhood via learning, conditioning, and socialization within the family and peer groups. A ruminative cognitive style, therefore, is seen as the result of a learning process related to the use of a passive coping style in response to negative affect. In line with this assumption, mothers’ depressive rumination and (negative) family functioning have been found to be positively related to children’s rumination [8,9]. Previous studies also found that parents who tend to discourage children’s emotional autonomy in the face of negative events may lead them to engage in rumination, increasing their vulnerability to depression [45]. Considering previous literature on the cognitive and emotional profile of parents of children with SLD, it is plausible to hypothesize that if parents do not feel they have adequate parental skills and/or they are exposed to increased parental challenges because of specific difficulties of their child, they might encounter difficulties in encouraging children’s emotional autonomy and positive coping styles, thus increasing the risk of rumination in their children. Nevertheless, to the best of our knowledge, no previous study directly assessed rumination in children with SLD and their parents. 

### 1.3. The Present Study

The main aim of the study was to evaluate rumination and its emotional/behavioral correlates (self-regulation, internalizing symptoms, peer relationships, behavioral profile) in children with SLD and in their parents compared to a sample of TD children and their parents. For the reasons reviewed above, it is plausible to hypothesize that students with SLD, and their parents, might experience higher levels of rumination compared to their TD peers. We also expect to observe higher levels of internalizing symptoms and difficulties with peers in children with SLD. 

Further, we explored potential associations between children’s and parents’ (mothers and fathers) ruminative style and between children’s tendency to ruminate and parents’ internalizing symptoms. Based on previous literature, we expect to find significant correlations between levels of rumination in children and their parents’ levels of ruminative and depressive symptoms. 

## 2. Method

### 2.1. Participants

The present study involved 50 Italian mother-tongue families, namely a child and his/her mother and father, for a total of 150 participants (50 children, 50 mothers, and 50 fathers). Twenty-five of the children received a diagnosis of SLD (mean age = 10.08 ± 1.15 years; 60% females), whereas the remaining 25 children were TD (mean age = 9.88 ± 0.53 years; 60% females). All the participants completed the study and there were no missing data. The two groups were balanced for gender and age, *t*(48) = 0.790, *p* = 0.434, as well as for the mothers’ education level (primary school; middle school; high school, or university), *χ*^2^(3) = 4.510, *p* = 0.211. A statistically significant difference was found for the fathers’ education level, *χ*^2^(3) = 8.659, *p* = 0.034, with fathers of children with SLD globally showing a lower education level.

Families in the clinical sample were recruited through word of mouth and through advice in clinical centers for the diagnosis of learning disorders. Children’s diagnosis was defined on the basis of a full neuropsychological assessment according to the International Classification of Diseases (ICD-10 code: F.81) and the standard Italian criteria included in the PARCC (Panel di Aggiornamento e Revisione della Consensus Conference) document, with scores below two SDs in standardized tests assessing reading, spelling, or math. Exclusionary criteria for diagnosis of SLD were neurological/sensorial disorders, socio-cultural disadvantage, or serious emotional/conduct problems. Twenty-four percent of the clinical sample had a diagnosis of dyslexia, 4% of dysorthographia (spelling), 16% of dyscalculia, and 56% had mixed learning disorders.

The TD group was recruited in two schools in the center of Italy. Inclusion criteria were the absence of a diagnosis of developmental disorder and never having been referred by teachers for suspected learning difficulties.

### 2.2. Measures

#### 2.2.1. Children

Kid Rumination Interview (KRI; ref. [8]). By the use of vignettes and cartoons, the KRI is well suited to assess rumination tendencies in children and early adolescents. The interview includes colored vignettes depicting four prototypical negative situations:

1. the child’s favorite toy has been broken;
2. the child’s t-shirt is dirty, and peers are teasing him/her;
3. parents are scolding their child because his/her bedroom is messy;
4. the child has just obtained a bad score at school.

The occurrence of each prototypical situation is depicted at three different times: a few hours after, before going to sleep, and the next day. For each of these three vignettes, the child is asked to report on a five-point Likert scale (from 1 = never to 5 = always), how often he/she happened to think over and over about the unpleasant event in a negative way. The questionnaire is composed of 12 items (three time points for each of the four prototypical situations), and the total score is derived from the sum of the scores across the four prototypical situations (higher scores indicate higher levels of rumination). When each vignette is presented, the child is asked whether he/she has experienced a similar event, with a yes/no response. The instrument includes a female and a male version based on the sex of the main character depicted in each vignette. For this study, four variables representing rumination (i.e., how often he/she happened to think over and over about the unpleasant event, mean of the three time points), one for each vignette, were calculated. Furthermore, a total rumination score based on the mean scores of the four vignettes was also calculated. The interview was originally developed in Italian and in the validation study, Cronbach’s alpha was 0.80 for the rumination about personal life dimension and 0.74 for the school-related rumination dimension, indicating good reliability [8]. 

Children’s Response Style Questionnaire (CRSQ [46]). The CRSQ is a self-report instrument consisting of 25 items divided into three subscales: rumination, (e.g., “*When I am sad, I think about how alone I feel*”), distraction (e.g., “*Watch TV or play video games so you do not think about how sad you are*”), and problem solving (e.g., “*Ask a friend/parent/teacher to help you solve your problem*”). Children are asked to indicate how often (from 0 = almost never to 3 = almost always) they engage in a specific behavior when they experience sadness. High scores indicate high levels of rumination, distraction, and poor problem-solving skills. This questionnaire has been derived from the adults’ Response Style Questionnaire [44]. The Italian version of the CRSQ showed Cronbach’s α > 0.80 for the three subscales [9].

Emotion Regulation Index for Children and Adolescents (ERICA; ref. [47]). The ERICA assesses three key emotional regulation components: (a) emotional control, whose items are indicative of emotion dysregulation or inappropriate emotional expression (e.g., “*I get angry when adults tell me what I can and cannot do*”); (b) emotional self-awareness, whose items are indicative of emotional awareness and modulation (e.g., “*I am a sad person*”); and (c) situational responsiveness, which includes items to assess empathy and affectivity that is appropriate to situational demands (e.g., “*I enjoy seeing others hurt or upset*”). Responses are given on a five-point Likert scale (from 1 = never to 5 = always). The internal consistency of the Italian version of the ERICA was strong, with Cronbach’s α > 0.78 for the subscales [8]. 

Multidimensional Anxiety Scale for Children (MASC; ref. [48]). MASC is a 39-item four-point Likert-style self-report scale aimed at assessing the presence of symptoms related to anxiety disorders in children and youth aged 8–19. The four main factors are: (a) physical symptoms (e.g., “*I have trouble breathing*”); (b) social anxiety (e.g., “*I am afraid the other children will laugh at me*”); (c) separation anxiety (e.g., “*I sleep next to someone from my family*”); and (d) harm avoidance (e.g., “*I do my utmost to obey my parents and teachers*”). Children can answer from “0 = never” to “3 = always”. The Italian version of the MASC has demonstrated good reliability [49]. 

#### 2.2.2. Parents

Strengths and Difficulties Questionnaire (SDQ; ref. [50]). The single-sided version of the Italian SDQ-parents version was administered to both mothers and fathers. The questionnaire includes 25 items describing positive and negative behavioral traits. The questionnaire’s subscales are emotional symptoms, conduct problems, hyperactivity–inattention, peer problems, and prosocial behavior, each with five items and a score ranging from 0 to 10. A scale of total difficulties, based on the four subscales assessing children’s difficulties, was also calculated (score range: 0–40). Respondents use a three-point Likert scale (0 = not true, 1 = somewhat true, and 2 = certainly true). Higher scores on the four subscales report difficulties, and a higher total score reflects more serious problems, whereas higher scores on the prosocial behavior subscale denote better social behavior. The scoring procedures are available online (www.sdqinfo.org). The Italian version of the SDQ has shown an acceptable internal consistency for all the scales and the total difficulties construct [51]. 

Rumination Response Scale (RRS; ref. [44]). The RRS is composed of 22 items that assess how often people engage in cognitive responses to depressed mood that are self-focused (e.g., “I think ‘Why do I react this way?’”), symptom-focused (e.g., “I think about ‘how hard it is to concentrate’”), and focused on the possible consequences and causes of one’s mood (e.g., “I think ’I will not be able to do my job if I do not snap out of this’”) on a four-point scale (from 1 = never to 4 = always). Higher scores indicate a higher level of depressive rumination. The Italian adaptation of the RRS has found the same good statistical properties as in the original version [52].

Penn State Worry Questionnaire (PSWQ; ref. [53]). The PSWQ is a 16-item self-report questionnaire commonly used to assess pathological worry in both clinical and non-clinical populations (e.g., “As soon as I finish one task, I start to worry about everything else I have to do”). Answers are given on a scale from 1 (“not at all typical of me”) to 5 (“very typical of me”). The internal reliability (Cronbach’s alpha = 0.92) and psychometric properties of the Italian version of the PSWQ have been demonstrated to be satisfactory [54]. 

Center for Epidemiological Studies—Depression (CES-D; ref. [55]). The CES-D is a self-report questionnaire that consists of 20 items evaluating the frequency of depressive symptoms over the previous week (e.g., “*I felt that everything I did was an effort*”) on a four-point scale: from “rarely or never” (if the symptom was observed for less than a day) to “often or all the time” (if the symptom was observed for 5/7 days). Total score ranges from 0 to 60 and standard cutoffs are >16 for mild depression and >23 for clinical depression. The psychometric properties of the Italian version of the CES-D have proven to be adequate [56].

State–Trait Anxiety Inventory (STAI; ref. [57]). The STAI consists of two 20-item scales that aim to measure state and trait anxiety; only the trait anxiety was used in this study. The STAI trait subscale asks respondents to rate how they feel “generally” (e.g., “*I lack self-confidence*”) on a four-point scale, from 1 (almost never) to 4 (almost always). The Italian adaptation of the test reported good reliability (Cronbach’s alpha = 0.91 for state anxiety and 0.85 for trait anxiety, respectively) [58].

### 2.3. Procedure

Parents filled in the questionnaires individually at their homes. The assessment battery for children required around 40 min to be completed; questionnaires were administered at school for the TD group, and at home or in the psychologist/speech-therapist’s study for the SLD group, by trainees in psychology. Each parent signed informed consent. The study protocol was approved by the Bioethics Committee of the University of Bologna (No. 2.5_29_11_16). 

### 2.4. Data Analysis

To investigate group differences in the children’s profile, multivariate analyses of variance (MANOVA) were performed with Group (SLD vs. TD) as between-subject factor, and the following dependent variables: subscales of the KRI, of the CRSQ, of the ERICA, of the MASC, and of the SDQ as filled in by mothers and fathers. For MANOVAs’ univariate results, the *p*-value for significance was set based on the Bonferroni correction. Independent sample t-tests were run in order to analyze group differences in the SDQ—total score for the two informants. Pearson correlations were then calculated to analyze the associations between the two variables representing children’s levels of rumination, namely total score on the KRI, the CRSQ-rumination scale, and the other CRSQ subscales, as well as on the ERICA, MASC, and SDQ scales. Taking into account the Bonferroni correction, the *p*-value for significance for these correlations was set at *p* < 0. 001.

Then, the parents’ profile was analyzed through independent groups (SLD vs. TD) t-tests run on RRS, PSWQ, CES, and STAI total scores, for both mothers and fathers, setting the *p*-value at 0.012 for the Bonferroni correction. Finally, correlations among parents’ variables and the two variables referring to their children’s rumination were calculated. In this case, considering the exploratory nature of these analyses, the Bonferroni correction was not considered [59].

## 3. Results

### 3.1. Children’s Profile

Descriptives of the variables included in the children’s profile, namely means and standard deviations for SLD and TD children, are represented in Table 1.

The MANOVA ran on the four vignettes of the KRI showed a significant multivariate effect of Group, *F*(4, 45) = 2.835, *p* = 0.035, *η*^2^ = 0.201, with a significant main effect of Group for the second vignette (i.e., teased by peers), *F*(1, 49) = 7.876, *p* = 0.007, *η*^2^ = 0.141, with children with SLD showing higher rumination scores for the scene in which the child is teased by peers. This result remained significant after the Bonferroni correction (*p* = 0.012). No main effect of Group emerged for the other three vignettes and for the KRI Total score *t*(48) = −0.857 *p* = 0.396, *d* = 0.24. No differences by group were found for scores on the CRSQ, *F*(3, 46) = 0.266, *p* = 0.850, *η*^2^ = 0.017, the ERICA, *F*(3, 46) = 0.896, *p* = 0.451, *η*^2^ = 0.055, and the MASC, *F*(4, 45) = 0.446, *p* = 0.774, *η*^2^ = 0.038, self-report questionnaires. Children with SLD reported to have experienced more often than their TD peers the negative events depicted in the fourth vignette (i.e., bad score at school; *χ*^2^(3) = 5.55, *p* < 0.018), but there were no differences for the other vignettes (all *p*s > 0.1). However, such difference in children’s experience with bad scores at school was no longer significant after the Bonferroni correction (*p* = 0.012).

Considering the SDQ filled in by mothers, a significant multivariate effect of Group emerged, *F*(5, 44) = 3.949, *p* = 0.005, *η*^2^ = 0.310, with children with SLD globally showing more negative symptoms. With univariate analyses, a significant main effect of Group emerged for the emotional symptoms, *F*(1, 49) = 5.809, *p* = 0.020, *η*^2^ = 0.108, conduct problems, *F*(1, 49) = 4.295, *p* = 0.044, *η*^2^ = 0.082, hyperactivity–inattention, *F*(1, 49) = 18.759, *p* < 0.001, *η*^2^ = 0.281, and peer problems *F*(1, 49) = 5.561, *p* = 0.022, *η*^2^ = 0.104 subscales; however, only the difference in hyperactivity–inattention remained significant after the Bonferroni correction (*p* = 0.01). Coherently, the SDQ total difficulties score, as assessed by mothers, was significantly higher for children with SLD, *t*(48) = 3.659, *p* = 0.001, *d* = 1.04. A partially different profile emerged from the SDQ filled in by the fathers, for which the multivariate effect of Group was only marginally significant, *F*(5, 44) = 2.193, *p* = 0.072, *η*^2^ = 0.200. A main effect of Group emerged, with children with SLD scoring higher on the hyperactivity–inattention subscale, *F*(1, 49) = 9.252, *p* = 0.004, *η*^2^ = 0.162, which remained statistically significant after Bonferroni correction, and marginally significant for the emotional symptoms, *F*(1, 49) = 3.317, *p* = 0.075, *η*^2^ = 0.065 and conduct problems, *F*(1, 49) = 3.491, *p* = 0.068, *η*^2^ = 0.068 subscales. Similar to mothers, fathers also referred a higher SDQ total score for children with SLD, *t*(48) = 2.572, *p* = 0.013, *d* = 0.73.

Correlation analysis investigating associations between children’s levels of rumination and the other examined variables, revealed only a significant result resistant to Bonferroni correction (*p* = 0.001), namely the link between the CRSQ-rumination score and the MASC subscale of social anxiety (*r* = 0.440, *p* = 0.001), indicating higher levels of rumination associated with higher levels of social anxiety.

### 3.2. Parents’ Profile

Descriptives for mothers’ and fathers’ variables, separated for SLD and TD groups, as well as the results of the *t*-tests analyzing group differences, are reported in Table 2. *T*-tests results showed higher levels of rumination for both mothers (only RRS) and fathers (both RRS and PSWQ) of children with SLD. Furthermore, mothers of children with SLD showed more symptoms of depression (CES-D), despite not scoring over the clinical cutoff. Mothers of children with SLD, as well as their fathers (tendency), also reported higher levels of anxiety (STAI). However, as shown by bold results in Table 2, only some of these group differences remained significant after the Bonferroni correction (*p* = 0.012): depressive rumination measured with the RRS for both mothers and fathers, and mothers’ levels of depressive symptoms (CES-D).

### 3.3. Associations between Children’s and Parents’ Variables

Finally, potential associations between parents’ variables and children’s levels of rumination were explored (Table 3). The KRI-Rumination total score was positively associated with the measures of rumination of both mothers and fathers, even though for fathers the correlation with RRS was only marginally significant. Moreover, a marginally significant correlation emerged for mothers’ scores on PSWQ and children’s scores on CRSQ-rumination. All the other correlations were not significant.

## 4. Discussion

The present study was aimed at assessing rumination and emotional/behavioral correlates in children with SLD and their parents, compared to a group of children with TD and their parents. 

First, results evidenced higher levels of rumination in children with SLD in response to a negative event in which the child is teased by peers, whereas no differences emerged in the other contexts, which were: child alone (broken toy), in the school context (bad score), or in family (the parents scold for the messy room). Despite not ruminating more on it, children with SLD reported to have experienced more often than their peers a negative event in the school context, but not in the other contexts. No group differences emerged in the total tendency to ruminate, suggesting that children with SLD, at the developmental age considered in the study (around 10 years old), do not have a general tendency to ruminate more compared to their TD peers but they may particularly suffer from the condition of being teased by peers (see [60] for a review). Although experiencing failures in school more often, children with SLD do not report engaging (more than their peers) in ruminative thoughts afterward, but they show repetitive and negative thinking when they face social failures. Previous studies have found that peer acceptance represents an important protective factor for children with SLD, who are at risk of developing feelings of unpopularity during the primary school years [61]. Interestingly, Kiuru et al. [62] found that high levels of peer acceptance uniquely contributed to ameliorating reading fluency after controlling for previous risks due to poor reading and other control variables. Furthermore, perspectives on social motivation have suggested that the role of supportive interpersonal relationships can serve as a resource for promoting students’ well-being and academic skills [63]. Based on the results of the present study, we might argue that children with SLD consider negative events in the school context in a similar way as their TD peers, probably because they are aware that bad scores can occur (e.g., they do not ruminate about bad scores more than others). On the counterpart, peer acceptance is for children with SLD a crucial variable for their well-being, because they need the support of their peers for accepting and overcoming their learning weaknesses. If they encounter failures in the social context, they have less positive coping strategies and might more frequently and intensively engage in repetitive thinking about the consequences of these negative events, leading to higher rumination levels. Rumination in children was found to be strongly related only to social anxiety, and this result fits with the observation that rumination in children with SLD mainly regards social context. Scores on the KRI modestly correlated with scores on a number of subscales of the DSQ (conduct problems, hyperactivity–inattention), but not with self-reported measures of anxiety or self-regulation. A plausible hypothesis is that at 10 years old, rumination has not fully developed as a stable trait and its relationships with other emotional variables is not fully structured. 

From their self-evaluation, children with SLD did not show higher levels of emotional dysregulation, anxiety, or physical symptoms compared to their TD peers, but, based on their parents’ evaluation, the profile of strengths and weaknesses of children with SLD was more compromised compared to that of TD peers. In particular, for both mothers and fathers, children with SLD are considered to have higher symptoms of hyperactivity–inattention and, although not significant after the Bonferroni correction, more emotional symptoms and conduct problems. Mothers of children with SLD also evidenced a tendency toward more difficulties in peer relationships compared to mothers of children with TD, although with no differences emerged as regards prosocial behavior. Overall, this pattern of results highlights a discrepancy between children’s self-evaluation and their parent’s evaluation. This trend has been found in previous studies, with absence of difference in emotional profile according to children self-evaluation and evidence of emotional disturbances in SLD when considering parents’ evaluation [6,64,65]. This is a point that would deserve future investigation as most studies draw their conclusions about emotional correlates of SLD based on parents’ reports. It might be that children underestimate or are not sufficiently aware of their emotional and behavioral difficulties, or that, based on social desirability, they tend to respond in such a manner as to avoid declaring their difficulties. On the other hand, as discussed below and in other studies [5,6,38], parents of children with SLD, having higher levels of parenting stress and/or internalizing symptoms, possibly due to the management of child’s difficulties and worries about their future, tend to overestimate children’s emotional discomfort, transposing their own discomfort on their perception of the child. 

With this regard, also in the present study, parents of children with SLD were found to have higher levels of depressive symptoms (mothers only), although not within the clinical range. Interestingly, they were also found to have higher levels of dispositional rumination, scoring higher than parents of TD children on the RRS. This is an important result that would deserve to be investigated in longitudinal studies, in light of previous evidence that suggests that a ruminative cognitive style in parents might be “learnt” by their offspring, particularly when entering into adolescence. In the present study, 10-year-old children presented only a tendency to greater tendencies to ruminate compared to TD peers, but the presence of higher trait rumination in their parents might represent a risk factor for children with SLD. 

As regards the association between children’s and parents’ variables, we found only modest (*p* < 0.05) correlations between parents’ and children’s rumination traits. Higher levels of rumination and worries in both fathers and mothers were positively correlated with scores on the KRI. There were no relationships between children’s rumination and parents’ scores on anxiety and depression scales. These results are in part in line with previous evidence of a relationship between parents’ and children’s levels of rumination [8,9], but do not replicate associations previously found with mothers’ depressive symptoms. 

The present study presents some limitations that need to be addressed in future investigations. First, the sample is relatively small and was not randomly selected, limiting the generalizability of results. In addition, the study included only parents and no other family members, such as siblings. Further, results from correlational analyses can only be interpreted as exploratory and do not allow defining eventual causal pathways. Longitudinal studies, as well as qualitative research, are strongly needed on this topic. Finally, in order to better understand the precise correlates of rumination in children with SLD, more fine-grained analyses of physiological activation are advocated. Ultimately, it is important to underline that the present study did not consider possible genetic influences, but only analyzed the cognitive and emotional/behavioral levels. It is possible that genetic heritance might have an effect not only on the transmission of SLD, as evidenced by family risk studies [39,40], but also on emotional aspects, and particularly rumination [66].

## 5. Conclusions

Despite these limitations, the present study offered an original contribution of rumination as a possible risk factor for children with SLD, particularly considering how they deal with peer acceptance and social failures. The study offers unique evidence of higher rumination tendencies after negative social events in children with SLD, associated with higher trait rumination in both of their parents. Considering that previous studies have found that rumination tends to increase with age and is strongly dependent on parents’ cognitive style, the present study has implications for clinical and educational settings. First, it seems to be important to support peer relationships in children with SLD; often the focus of supports for this population is the learning domain (didactic strategies, interventions in reading/writing skills), but the present study suggests that children with SLD give major importance to social support and acceptance, so it would be necessary to favor positive peer relationships in the scholastic and extra-scholastic settings. Second, the evidence of emotional discomfort in parents leads to a recommendation that teachers and clinicians dedicate more attention to psychological well-being and positive parenting in mothers and fathers of children with SLD. Within a family-centered approach [67], the concept of care in the children should encompass caregivers.

## Figures and Tables

**Table 1:** Descriptives for children’s variables considering the special learning disorder (SLD) and typically developing (TD) groups.

Variable	Mean (SD) SLD *n* = 25	Mean (SD) TD *n* = 25
KRI–Vignette 1 (broken toy)	7.64 (2.55)	7.84 (2.36)
KRI–Vignette 2 (teased by peers)	**8.84 (2.90)**	6.72 (2.42) *
KRI–Vignette 3 (scolded by parents)	6.72 (3.16)	7.04 (2.39)
KRI–Vignette 4 (bad score at school)	8.04 (2.86)	7.88 (2.09)
KRI-Rumination total score	31.24 (8.24)	29.48 (6.12)
CRSQ–Rumination	12.40 (6.32)	14.04 (6.50)
CRSQ–Distraction	8.56 (4.25)	8.72 (4.40)
CRSQ–Problem Solving	7.20 (3.44)	7.48 (3.65)
ERICA–Emotional control	19.72 (4.52)	18.16 (4.51)
ERICA–Emotional self-awareness	14.28 (3.06)	15.16 (2.75)
ERICA–Situational responsiveness	12.64 (2.04)	12.72 (1.49)
MASC–Physical symptoms	13.72 (5.22)	11.84 (4.54)
MASC–Social anxiety	9.72 (4.48)	8.60 (5.37)
MASC–Separation anxiety	18.16 (3.30)	17.88 (3.13)
MASC–Harm avoidance	10.44 (5.37)	9.68 (4.71)
SDQ mother–Emotional symptoms	3.48 (2.38)	2.00 (1.94) *
SDQ mother–Conduct problems	2.24 (1.59)	1.40 (1.26) *
SDQ mother–Hyperactivity–inattention	**4.76 (2.42)**	2.24 (1.61) *
SDQ mother–Peer problems	2.00 (2.00)	0.92 (1.11) *
SDQ mother–Prosocial behavior	8.00 (1.61)	8.60 (1.26)
SDQ mother–Total Difficulties	12.48 (6.60)	6.56 (4.67) *
SDQ father–Emotional symptoms	2.68 (1.82)	1.68 (2.06)
SDQ father–Conduct problems	2.08 (1.58)	1.32 (1.28)
SDQ father–Hyperactivity–inattention	**4.40 (1.94)**	2.72 (1.97) *
SDQ father–Peer problems	1.92 (1.85)	1.32 (1.38)
SDQ father–Prosocial behavior	8.40 (1.22)	7.84 (2.70)
SDQ father–Total Difficulties	11.08 (5.67)	7.04 (5.43)

Note: SLD = Specific Learning Disorders; TD = Typical Development; KRI = Kid Rumination Interview; CRSQ = Children’s Response Style Questionnaire; ERICA = Emotion Regulation Index for Children and Adolescents; MASC = Multidimensional Anxiety Scale for Children; SDQ = Strengths and Difficulties Questionnaire. * Significant group difference based on univariate MANOVAs’ results; in bold, differences that remained statistically significant after Bonferroni correction.

**Table 2:** Descriptives for mothers’ and fathers’ variables and results of t-tests for group differences.

Group	Variable	Mean (SD) SLD *n* = 25	Mean (SD) TD *n* = 25	*t*(48), *p*	Cohen’s *d*
Mothers *n* = 25	Rumination Response Scale	41.52 (6.72)	35.64 (9.10)	**2.599, *p* = 0.012**	0.74
	Penn State Worry Questionnaire	43.24 (11.82)	38.28 (12.15)	1.463, *p* = 0.150	0.41
	Center for Epidemiological Studies—Depression	12.56 (6.40)	7.88 (5.06)	**2.867, *p* = 0.006**	0.81
	State–Trait Anxiety Inventory	39.52 (9.24)	34.44 (7.98)	2.080, *p* = 0.043	0.59
Fathers *n* = 25	Rumination Response Scale	38.08 (6.08)	30.36 (10.65)	**3.147, *p* = 0.003**	0.89
	Penn State Worry Questionnaire	40.96 (11.21)	33.92 (8.81)	2.468, *p* = 0.017	0.70
	Center for Epidemiological Studies—Depression	12.16 (7.45)	9.04 (4.93)	1.747, *p* = 0.087	0.49
	State–Trait Anxiety Inventory	42.80 (7.98)	38.32 (8.01)	1.981, *p* = 0.053	0.56

Note. In bold, *t*-tests that remained statistically significant after the Bonferroni correction.

**Table 3:** Pearson correlations between parents’ variables and the variables referring to their children’s levels of rumination.

Group	Variable	Children’s KRI-Rumination *n* = 50	Children’s CRSQ-Rumination *n* = 50
Mothers *n* = 50	Rumination Response Scale	0.286 *	0.040
	Penn State Worry Questionnaire	0.289 *	0.268 ^a^
	Center for Epidemiological Studies—Depression	0.122	0.088
	State–Trait Anxiety Inventory	0.237	0.101
Fathers *n* = 50	Rumination Response Scale	0.269 ^a^	0.145
	Penn State Worry Questionnaire	0.325 *	0.231
	Center for Epidemiological Studies—Depression	0.026	0.085
	State–Trait Anxiety Inventory	0.215	0.163

* *p* < 0.05; ^a^ *p* ≤ 0.060. KRI = Kid Rumination Interview; CRSQ = Children’s Response Style Questionnaire.
